# ^18^F-PSMA-1007 versus ^18^F-FDG PET/CT in the detection of hepatocellular carcinoma

**DOI:** 10.3389/fonc.2025.1711398

**Published:** 2025-12-08

**Authors:** Xiaoli Mei, Yanpeng Li, Shasha Xu, Xiaoping Shang, Bing Cheng, Xiaobo Niu, Xiaoting Liu, Yan Liu, Xinya Cheng, Xingmin Han, Ruihua Wang

**Affiliations:** 1 Department of Nuclear Medicine, The First Affiliated Hospital of Zhengzhou University, Henan Medical Key Laboratory of Molecular Imaging, Zhengzhou, China; 2 Department of Medical Records, The First Affiliated Hospital of Zhengzhou University, Zhengzhou, China; 3 Faculty of Arts and Social Science, Hong Kong Baptist University, Hong Kong, Hong Kong SAR, China

**Keywords:** 18F-PSMA-1007, 18F-FDG, hepatocellular carcinoma (HCC), prostate specific membrane antigen (PSMA), diagnostic performances

## Abstract

**Background:**

Prostate-specific membrane antigen (PSMA) is expressed in hepatocellular carcinoma (HCC). Recently, ^18^F-PSMA-1007 has been used for tumor imaging in positron emission tomography/computed tomography (PET/CT). This study aimed to compare the diagnostic performances of ^18^F-PSMA-1007 PET/CT and ^18^F-FDG PET/CT in HCC, and to assess factors associated with the sensitivity of ^18^F-PSMA-1007 PET/CT in detecting HCC and intrahepatic tumor lesions.

**Materials and Methods:**

Forty-four patients with suspected HCC undergoing both ^18^F-FDG and ^18^F-PSMA-1007 PET/CT were prospectively enrolled. Two experienced nuclear medicine physicians independently interpreted imaging results. The mean standardized uptake values (SUV_mean_) were measured in the intrahepatic lesions (T), liver background (L), abdominal aorta (A), and right medial gluteal muscle (M), respectively. The tumor-to-background ratio (T/L, T/A, T/M) was then calculated as the SUV_mean_ of the intrahepatic lesion (T) divided by the SUV_mean_ of the background regions (L, A, M).

**Results:**

Sixty-two intrahepatic lesions in 41 patients with HCC were finally involved in the statistical analysis. ^18^F-PSMA-1007 PET/CT demonstrated higher sensitivity than ^18^F-FDG PET/CT in detecting HCC patients (85.4% *vs.* 61.0%, *P* = 0.041), particularly in identifying well- or moderately differentiated HCC patients (92.9% *vs.* 14.3%, *P* = 0.003). ^18^F-PSMA-1007 PET/CT showed a higher sensitivity than ^18^F-FDG PET/CT in detecting intrahepatic HCC lesions (82.3% *vs.* 50.0%, *P* = 0.001), including in small (≤ 2 cm in diameter; 62.5% *vs.* 25.0%, *P* = 0.049) and well- or moderately differentiated (88.9% *vs.* 14.8%, *P* < 0.001) lesions. The sensitivity of ^18^F-PSMA-1007 PET/CT was associated with tumor size (*P* = 0.005). The SUV_mean_ values for the intrahepatic lesions (T) and liver background (L) from ^18^F-PSMA-1007 PET/CT were significantly higher compared with those from ^18^F-FDG PET/CT (both *P* < 0.001). Background uptake in the abdominal aorta (A) and right medial gluteal muscle (M) for ^18^F-PSMA-1007 was significantly lower than that for ^18^F-FDG (both *P* < 0.001). T/L, T/A and T/M values from ^18^F-PSMA-1007 were significantly higher than those from ^18^F-FDG PET/CT (all *P* < 0.001).

**Conclusions:**

^18^F-PSMA-1007 PET/CT exhibits higher sensitivity than ^18^F-FDG PET/CT for detecting HCC and has lower background uptake in blood and muscle tissues. The sensitivity of ^18^F-PSMA-1007 is correlated mainly with tumor size.

## Introduction

Liver cancer is the sixth most common cancer worldwide and the fourth most prevalent cause of cancer-related mortality globally, and hepatocellular carcinoma (HCC) is the most common type of liver cancer, accounting for about 90% of cases (1, 2). Early diagnosis of new or recurrent HCC in at-risk patients offers the most favorable opportunity for effective treatment and enhances long-term disease-free survival (3). Unlike many other malignant tumors, HCC can be diagnosed by imaging based on non-invasive criteria without confirmatory pathology (4). Therefore, imaging plays a critical role in the detection and diagnosis of HCC (5). To date, imaging in HCC mainly relies on computed tomography (CT) and magnetic resonance imaging (MRI) (6, 7), both of which rely on change in size, contrast enhancement and wash-out characteristics to diagnose lesions suspicious for HCC (8). Anatomic imaging can be limited by atypical imaging characteristics, reduced resolution in small lesions and is complicated by altered parenchymal architecture on a background of significant liver cirrhosis and patient factors including body habitus and previous treatment. Molecular imaging offers the advantage of detecting functional abnormalities that often precede anatomical changes identifiable through morphological imaging in oncological diseases (9). Among molecular imaging techniques, Fluorine-18 fluorodeoxyglucose (^18^F-FDG) positron emission tomography/computed tomography (PET/CT) has been extensively studied in HCC patients (10). However, ^18^F-FDG has a limited role in the evaluation of patients with HCC, as this tumor type exhibits ^18^F-FDG avidity in fewer than half of cases, particularly in well-differentiated HCC (11).

Prostate-specific membrane antigen (PSMA), also referred to as glutamate carboxypeptidase type II, is a transmembrane protein encoded by the gene FOLH1, first identified in prostate cancer cells in 1987 (12, 13). It has been observed that PSMA is not exclusively expressed by prostate cancer cells; rather, it is also present on the surface of various cancer cell types and neovascular endothelial cells associated with different solid tumors (14). Given that PSMA is overexpressed by neovascular endothelial cells in numerous malignancies, including HCC, this provides a rationale for employing PET/CT or PET/MRI with PSMA-radioligands in tumors exhibiting low ^18^F-FDG uptake, thereby evaluating molecular pathways beyond glucose metabolism. This study aimed to comparatively assess the diagnostic performances of ^18^F-PSMA-1007 PET/CT and ^18^F-FDG PET/CT in HCC and to assess factors associated with the sensitivity of ^18^F-PSMA-1007 PET/CT in detecting HCC and intrahepatic tumor lesions.

## Materials and methods

### Patients

This is a *post-hoc* analysis of a prior prospective study conducted at the First Affiliated Hospital of Zhengzhou University from June 2023 to October 2024. Forty-four patients with suspiciously incipient HCC determined by clinical manifestations and conventional imaging techniques (CT, MRI and ultrasound) were included in this study. They underwent both ^18^F-FDG and ^18^F-PSMA-1007 PET/CT examinations with an interval of one day before surgical treatment. According to Standardization for diagnosis and treatment of hepatocellular carcinoma (2022 edition), for patients who underwent surgery or biopsy, the definitive diagnosis was confirmed by pathology. In cases where patients received transarterial chemoembolization (TACE), the HCC diagnosis was based on a specific imaging pattern characterized by hyperenhancement in the arterial phase and washout in the venous or delayed phase, as observed on contrast-enhanced CT or MRI in the context of liver cirrhosis (3, 15). The inclusion criteria were as follows: (1) HCC diagnosed by pathology; (2) patients exhibiting typical imaging features of HCC on contrast-enhanced CT or MRI who had not undergone surgery or biopsy; (3) signed informed consent; and (4) willingness to accept follow-up. Exclusion criteria included: (1) patients who had received local or systemic treatment; (2) patients with concurrent other types of tumors; and (3) patients with mental disorders who were unable to cooperate with the examination. This study received approval from the Ethics Committee (2022-KY-0482).

### Radiopharmaceutical

^18^F-PSMA-1007 precursor, cassettes, and reagents for the synthesis of ^18^F-PSMA-1007 were procured from ABX Advanced Biochemical Compounds (Radeberg, Germany). The synthesis of ^18^F-PSMA-1007 was conducted using the HM-20 cyclotron and CFN-100 synthesis module from Sumitomo Corporation (Japan). The synthesis of ^18^F-FDG was carried out according to the methodology outlined by Gallagher et al. (16). The radiotracer was achieved with a radiochemical purification yield exceeding 98%, as determined by radio-thin-layer chromatography and high-performance liquid chromatography analysis. All products were prepared utilizing advanced technology under aseptic and pyrogen-free conditions.

### PET/CT imaging

Whole-body ^18^F-FDG and ^18^F-PSMA-1007 PET/CT scans were obtained on the same PET/CT scanner [Biograph TruePoint64 (52) ring, Siemens, Germany]. Whole-body static ^18^F-FDG PET/CT scans were obtained as a routine procedure. Whole-body ^18^F-PSMA-1007 PET/CT images were acquired 60 min after tracer injection, as referenced by Kuten et al. (17). The doses of ^18^F-FDG and ^18^F-PSMA-1007 were determined according to patient weight: < 60 kg (200 MBq), 61∼90 kg (250 MBq), > 90 kg (300 MBq). Low-dose CT (120 keV and 100 mAs per section, pitch of 0.8 mm) scans were obtained for attenuation correction and image fusion. PET images were acquired in the 3D mode. After attenuation correction of PET images with CT data, and whole-body PET and CT images were generated through iterative reconstruction.

### Image analysis

^18^F-FDG and ^18^F-PSMA-1007 PET/CT images were assessed by two experienced, certified nuclear medicine physicians blinded to other imaging and pathology results. All scans were anonymized and independently reviewed by each physician to mitigate subjective bias. Concordance in cases with differing results was achieved through consensus. The mean standardized uptake values (SUV_mean_) of primary lesions (T) was obtained by outlining a volume of interest that included the lesion in all three planes in ^18^F-FDG and ^18^F-PSMA-1007 PET/CT images. Moreover, regions of interest with a diameter of 1 cm were drawn from lesion-free liver tissue (L), the abdominal aorta (A), and the right medial gluteal muscle (M) for SUV_mean_ measurements. Using these three background SUV_mean_ values, tumor-to-normal liver parenchyma (T/L), tumor-to-abdominal aorta (T/A), and tumor-to-gluteal muscle (T/M) ratios were calculated separately. A lesion was considered to be positive on the basis of the visual judgment of elevated uptake in the tumor tissue by 2 experienced nuclear medicine physicians independently, supported by the calculation of the T/L of ^18^F-FDG and ^18^F-PSMA-1007, respectively.

### Statistical analysis

SPSS version 26.0 statistical software (IBM Corp., Armonk, NY) was employed for data analysis. Categorical variables were presented as frequency and percentage (%), and continuous variables conforming to normal distribution were expressed as mean ± standard deviation (SD), whereas continuous variables not conforming to normal distribution were represented as median [interquartile range (IQR)]. The McNemar’s test, Chi-square test, and Fisher’s exact test were employed to compare categorical variables. The Paired t-test was utilized to compare dependent variables that followed a normal distribution. Two-tailed *P* < 0.05 was considered statistically significant.

## Results

### Patient characteristics

**Figure 1:**
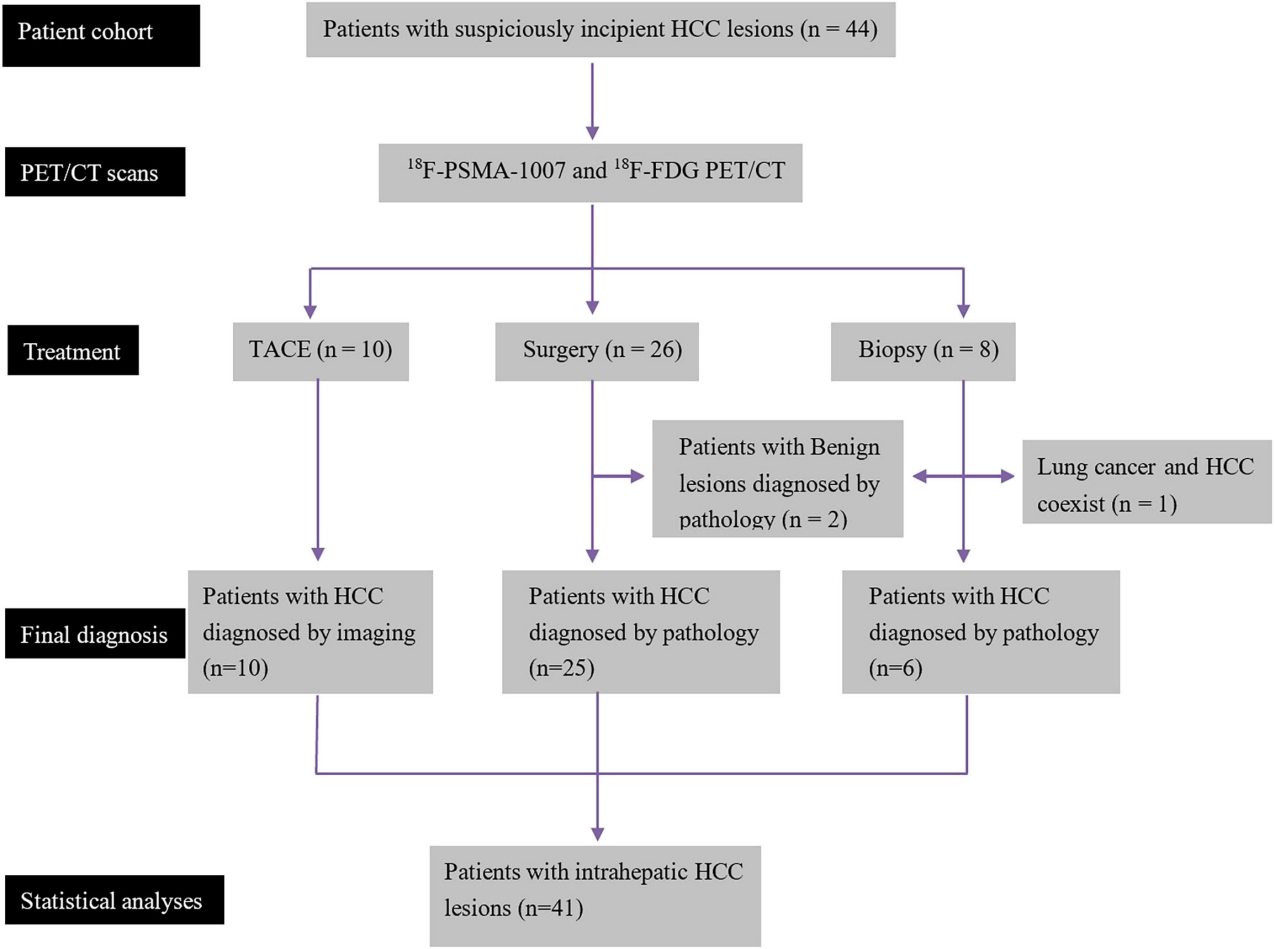
Study flowchart (n = number of patients).

Forty-four patients were included in the current study, comprising 26 individuals who underwent hepatic surgery, 10 administered TACE, and eight who received biopsy only. With the exception of two patients diagnosed with benign hepatic nodules and one patient with lung cancer and hepatocellular carcinoma coexisting, the remaining 41 patients were diagnosed with HCC. Thus, 41 HCC patients with 62 intrahepatic lesions were ultimately included in the statistical analysis. The study flowchart is presented in **Figure 1**. According to microvascular invasion (MVI) number and distribution, 8, 12 and 5 patients were categorized into the M0 (no MVI), M1 (≤ 5 MVI in adjacent liver tissue ≤ 1 cm away from the HCC), and M2 (> 5 MVI or MVI in adjacent liver tissue > 1 cm away from the HCC) groups, respectively. According to histologic grade of HCC (18), 2, 12 and 11 patients were categorized into histologic grade I, II and III, respectively. The general characteristics of the 41 HCC patients are summarized in **Table 1**.

**Table 1:** Characteristics of the included HCC patients.

General characteristics	n =41	%
Age (years)	52.8 ± 7.8	
Gender (male)	34	82.9
HBsAg (+)	37	90.2
Anti-HCV (+)	3	7.3
Cirrhosis	32	78.0
AFP (> 10 ng/mL)	32	78.0
Tumor number		
Solitary tumor	31	75.6
Multiple tumors	10	24.4
Histologic grade		
I	2	4.9
II	12	29.3
III	11	26.8
MVI		
M0	8	19.5
M1	12	29.3
M2	5	12.2

Ten HCC patients whose diagnosis was based on non-invasive criteria underwent TACE and six HCC patients whose diagnosis relied on biopsy instead of hepatic surgery and, therefore, had no complete pathological data. HBsAg, hepatitis B surface antigen; Anti-HCV, anti-hepatitis C virus antibody; AFP, α-fetoprotein; MVI, microvascular invasion.

### Comparison of ^18^F-PSMA-1007 with ^18^F-FDG in patient-based analysis

**Figure 2:**
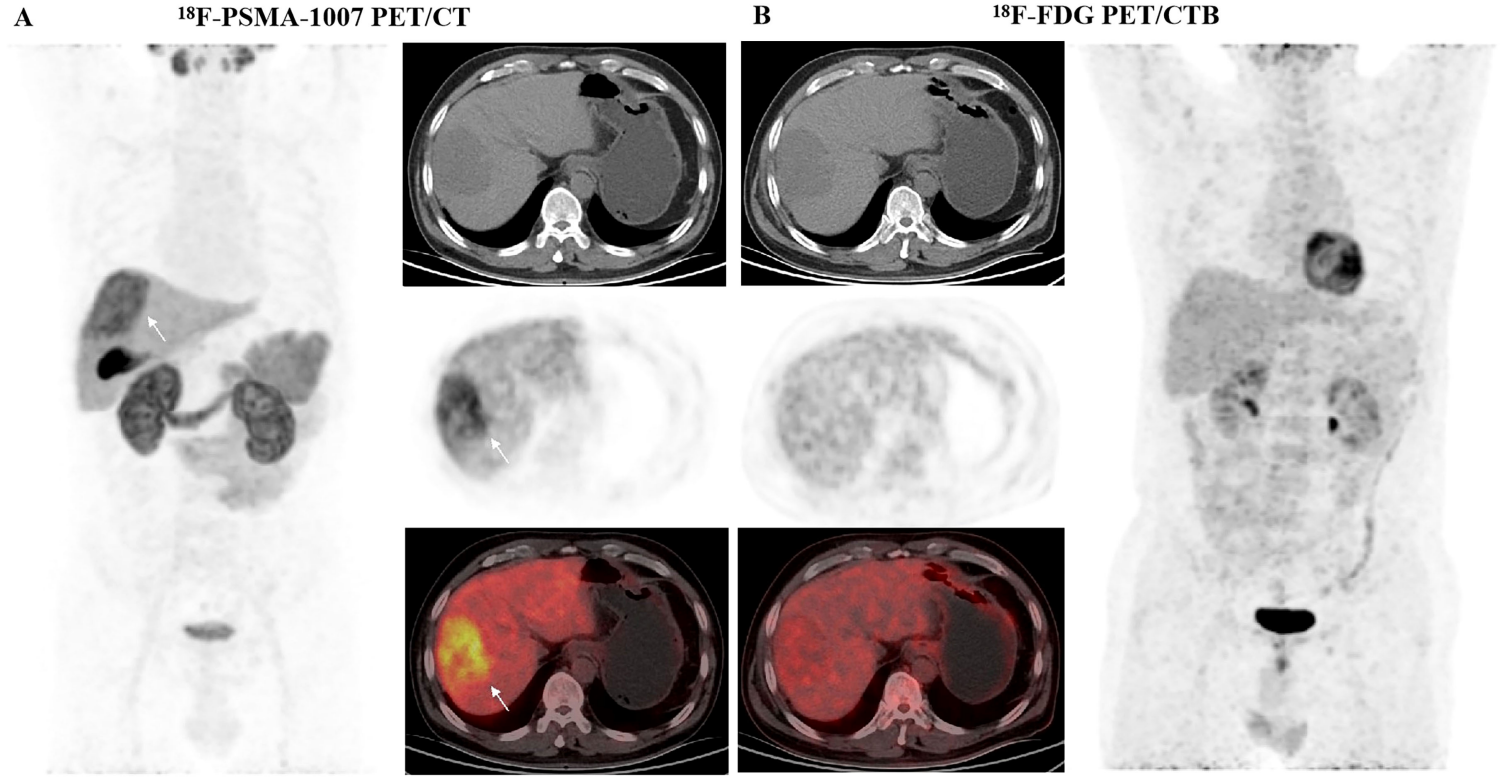
PET/CT images in a 54-year-old male patient with well-differentiated HCC. **(A)**^18^F-PSMA-1007 PET/CT revealed a strongly PSMA-avid lesion (white arrows, SUV_max_ = 20.3, T/L = 2.1) that was pathologically confirmed as well-differentiated HCC. **(B)**^18^F-FDG PET/CT showed no elevated uptake in the low-density mass of the liver right lobe (SUV_max_ = 4.6; T/L = 1.2).

Among the 41 HCC patients, 20 were positively identified by both ^18^F-PSMA-1007 and ^18^F-FDG PET/CT, 15 were positively identified by ^18^F-PSMA-1007 alone, and 1 was not positively identified by either imaging tracer. The sensitivity of ^18^F-PSMA-1007 PET/CT in detecting HCC patients was superior compared with ^18^F-FDG PET/CT (85.4% *vs.* 61.0%, *P* = 0.041, **Table 2**). ^18^F-PSMA-1007 PET/CT demonstrated greater sensitivity than ^18^F-FDG PET/CT in the detection of well- or moderately differentiated HCC patients (*P* = 0.003). ^18^F-PSMA-1007 PET/CT detected 13 of the 14 well- or moderately differentiated HCC patients, whereas ^18^F-FDG PET/CT detected only 2 in these cases (**Figure 2**). The sensitivity of ^18^F-FDG PET/CT was associated with histologic grade (*P* < 0.001), while that of ^18^F-PSMA-1007 PET/CT was not correlated with those clinical and pathological features, such as cirrhosis, AFP levels, tumor number, MVI, or histologic grade (all *P* > 0.05). These findings suggested that ^18^F-PSMA-1007 PET/CT was more sensitive than ^18^F-FDG PET/CT in the detection of well- or moderately differentiated HCCs.

**Table 2:** Sensitivities of ^18^F-FDG and ^18^F-PSMA-1007 PET/CT in patient-based analysis.

Patient characteristics	No.	^18^F-PSMA-1007		^18^F-FDG		*P* between 2 tracers
		Positive (%)	*P*	Positive (%)	*P*	
ALL	41	35 (85.4%)		25 (61.0%)		0.041*
Clinical features						
Cirrhosis	32	27 (84.4%)	1	19 (59.4%)	1	0.077
Non-cirrhosis	9	8 (88.9%)		6 (66.7%)		0.625
AFP (ng/mL)						
≤10	9	8 (88.9%)	1	5 (55.6%)	0.717	0.375
> 10	32	27 (84.4%)		20 (62.5%)		0.118
Tumor number						
Solitary tumor	31	25 (80.6%)	0.66	20 (64.5%)	1	0.302
Multiple tumors	10	9 (90.0%)		6 (60.0%)		0.375
MVI						
M0 + M1	20	17 (85.0%)	0.25	9 (45.0%)	0.322	0.057
M2	5	3 (60.0%)		4 (80.0%)		1
Histologic grade						
I + II	14	13 (92.9%)	0.13	2 (14.3%)	<0.001*	0.003*
III + IV	11	7 (63.6%)		11 (100%)		–

Sixteen HCC patients had no complete pathological data. No., number of patients; *, statistically significant; AFP, a-fetoprotein; MVI, microvascular invasion.

### Comparison of ^18^F-PSMA-1007 with ^18^F-FDG in lesion-based analysis

^18^F-PSMA-1007 PET/CT showed a better sensitivity in detecting intrahepatic lesions compared with ^18^F-FDG PET/CT (82.3% *vs.* 50.0%, *P* = 0.001, **Table 3**). ^18^F-PSMA-1007 PET/CT was more sensitive than ^18^F-FDG PET/CT in detecting small intrahepatic lesions (≤ 2 cm in diameter) (*P* = 0.049) and well- or moderately differentiated intrahepatic lesions (*P* < 0.001), but there were no significant sensitivity differences between the 2 tracers in the detection of HCCs > 2 cm in diameter (both *P* > 0.05) and poorly- differentiated or undifferentiated HCCs (*P* > 0.05). The sensitivities of ^18^F-PSMA-1007 PET/CT were significantly related to the size of intrahepatic lesions (both *P* < 0.05). These findings indicated that ^18^F-PSMA-1007 PET/CT was more sensitive than ^18^F-FDG PET/CT in the detection of small and well- or moderately differentiated HCCs, and ^18^F-PSMA-1007 PET/CT was more sensitive in detection of big intrahepatic lesions.

**Table 3:** Sensitivities of ^18^F-PSMA-1007 and ^18^F-FDG PET/CT in lesion-based analysis.

Lesion characteristics	No.	^18^F-PSMA-1007		^18^F-FDG		*P* between 2 tracers
		Positive lesions (%)	*P*	Positive lesions (%)	*P*	
All	62	51 (82.3)		31 (50.0)		0.001*
Diameter (cm)						
≤ 2	24	15 (62.5)	0.005*	6 (25.0)	0.003*	0.049*
> 2, ≤ 5	12	11 (91.7)		6(50.0)		0.125
> 5	26	25(96.2)		19 (73.1)		0.070
Histologic grade						
I + II	27	24 (88.9)	0.125	4 (14.8)	<0.001*	<0.001*
III + IV	13	9 (69.2)		12 (92.3)		0.375

Twenty-two lesions had no pathological data; No., number of lesions; *, Statistically significant.

### Uptake intensities of ^18^F-PSMA-1007 an^18^F-FDG in HCC(patient-based analysis)

**Figure 3:**
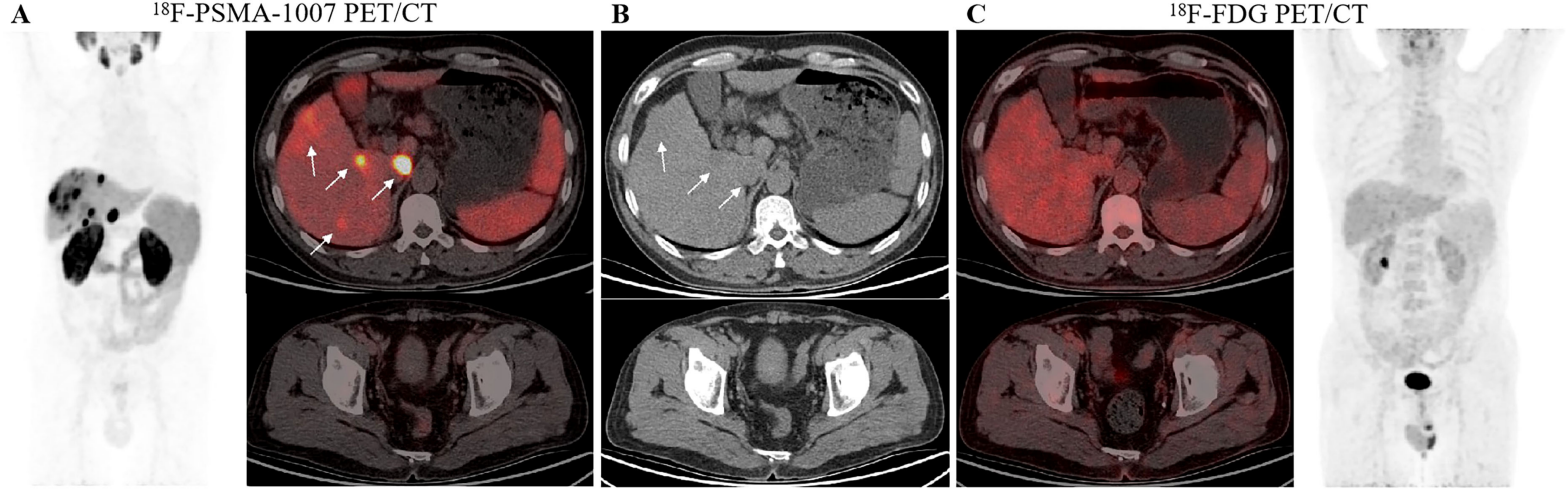
Comparison of ^18^F-PSMA-1007 and ^18^F-FDG PET/CT imaging in a 63-year-old man with HCC (arrow indicates lesion). **(A)** Maximum-intensity projections (MIP) of PET using ^18^F-PSMA-1007 and transversal fusion images of primary hepatocellular carcinoma and right medial gluteal muscle (T: 71.6, L: 7.6, T/L: 9.4; A: 1.1, T/A: 65.1; M: 0.5, T/M: 143.2). **(B)** Transverse CT images revealed low-density masses in the right lobe of the liver and right medial gluteal muscle **(C)** MIP of PET using ^18^F-FDG and the radiation uptake of HCC and right medial gluteal muscle (T:3.4, L:3.5, T/L: 1.0; A: 2.1, T/A: 1.6; M: 1.1, T/M: 3.1).

Among the 41 HCC patients, the uptake of ^18^F-FDG and ^18^F-PSMA-1007 in intrahepatic lesions, normal liver parenchyma, the abdominal aorta, and the right medial gluteal muscle was assessed, respectively (**Table 4**). The SUV_mean_ of intrahepatic lesions (T) for ^18^F-FDG and ^18^F-PSMA-1007 PET/CT were 3.56 (2.21-7.98) and 21.42 (12.76-44.71), respectively. The SUV_mean_ of normal liver parenchyma (L) for ^18^F-FDG and ^18^F-PSMA-1007 PET/CT were 2.33 (2.02-3.14) and 7.83 ± 2.32. A statistically significant difference was observed among T, L value between ^18^F-FDG and ^18^F-PSMA-1007 (all *P* < 0.001). The values for T, L with ^18^F-PSMA-1007 PET/CT were significantly higher than those for ^18^F-FDG (**Figure 3**). The SUV_mean_ of the abdominal aorta (A) for ^18^F-FDG and ^18^F-PSMA-1007 PET/CT were 1.65 (1.32-1.9) and 1.12 (0.83-1.54), respectively. The SUV_mean_ for the right medial gluteal muscle (M) in ^18^F-FDG and ^18^F-PSMA-1007 PET/CT were 0.89 (0.83-1.10) and 0.52 (0.41-0.59), respectively. Notably, both A and M values for ^18^F-PSMA-1007 PET/CT were significantly lower than those for ^18^F-FDG in each patient (all *P* < 0.001). When comparing the T/A, T/M, and T/L ratios, T/A, T/M, and T/L exhibited statistically significant differences between ^18^F-FDG and ^18^F-PSMA-1007 (all *P* < 0.001). The T/A, T/G, and T/L ratios for ^18^F-PSMA-1007 were significantly higher than those for ^18^F-FDG.

**Table 4:** Uptake Intensities of ^18^F-PSMA-1007 and ^18^F-FDG in HCC.

SUV_mean_	^18^F-PSMA-1007	^18^F-FDG	*P*
T	21.42 (12.76-44.71)	3.56 (2.21-7.98)	<0.001*
L	7.83 ± 2.32	2.33 (2.02-3.14)	<0.001*
T/L	3.42 (2.14-6.22)	1.33 (1.1 - 2.98)	<0.001*
A	1.12 (0.83-1.54)	1.65 (1.32-1.9)	<0.001*
T/A	25.12 (10.9 - 46.73)	2.42 (1.83- 6.71)	<0.001*
M	0.52 (0.41-0.59)	0.89 (0.83-1.10)	<0.001*
T/M	56.9 (25.7 - 98.84)	5.2 (3.85 - 112.1)	<0.001*

T, SUV_mean_ of intrahepatic lesions; L, SUV_mean_ of normal liver parenchyma; A, SUV_mean_ of abdominal aorta; M, SUV_mean_ of right medial gluteal muscle; *, statistically significant.

## Discussion

Preclinical studies have suggested that PSMA may regulate tumor cell invasion and neo-angiogenesis by modulating integrin signal transduction in endothelial cells (19). Over the past five years, several studies have evaluated the diagnostic performance of ^68^Ga-PSMA-11 for detecting HCC lesions in newly diagnosed patients as well as in individuals previously treated with various modalities of local or systemic therapy (20–25). The use of ^18^F-labeled PSMA-targeting radiopharmaceuticals offers several advantages, including large-scale production, reduced costs, and high-quality imaging due to lower positron energy and an extended half-life (26). Preclinical studies indicated that lesions expressing PSMA exhibited a higher affinity and internalization rate for ^18^F-PSMA-1007 compared to ^68^Ga-PSMA-11, resulting in increased radioactivity uptake (27, 28). In this prospective study, we investigated the contribution of ^18^F-PSMA-1007 PET/CT to the diagnostic value in newly diagnosed HCC. The principal findings of this study indicated that^18^F-PSMA-1007 demonstrated higher sensitivity in detecting HCC and provided better tumor-to-background contrast in blood and muscle tissues compared to^18^F-FDG. The sensitivity of ^18^F-PSMA-1007 is correlated mainly with tumor size.

In accordance with prior research indicating sensitivities ranging from 40% to 68%, the sensitivity of ^18^F-FDG PET/CT for the detection of HCC was determined to be 61.0% in the present study (29). Comparatively, ^18^F-PSMA-1007 PET/CT demonstrated superior sensitivity (85.4%) for detecting HCC patients. Of note, ^18^F-PSMA-1007 PET/CT exhibited a relatively higher sensitivity in identifying well- or moderately differentiated HCC patients (13 of 14, histologic grade I or II) compared to ^18^F-FDG PET/CT (2 of 14). Furthermore, ^18^F-PSMA-1007 PET/CT was more sensitive than ^18^F-FDG PET/CT in detecting well- or moderately differentiated intrahepatic lesions (88.9% *vs.* 14.8%). ^18^F-FDG metabolism may exhibit significant variability in HCC contingent upon its differentiation. The poor sensitivity of ^18^F-FDG PET/CT in detecting low-grade HCC is probably related to enhanced glucose-6-phosphatase activity causing the dephosphorylation of ^18^F-FDG-6-PO4, which is therefore not trapped in HCC cells, resulting in false-negative results (29, 30). It is well known that neo-angiogenesis serves as a crucial factor in tumor growth, PSMA is overexpressed in the neovascular endothelial cells of HCC (31). Literature indicates that nearly 95% of HCCs express PSMA in tumor neovascularization (32–34); moderate to high levels of PSMA positivity are evident in approximately 80% of HCC cases (35), whereas completely PSMA-negative HCCs constitute a minority (4.1%). Therefore, ^18^F-PSMA-1007 PET/CT appears to be a promising new approach for the detection of intrahepatic HCC lesions with higher sensitivity compared with ^18^F-FDG PET/CT.

The sensitivity of ^18^F-FDG PET/CT was associated with histologic grade and ^18^F-FDG PET/CT exhibited a relatively lower sensitivity in identifying well- or moderately differentiated HCCs than poorly- differentiated or undifferentiated HCCs (14.3% *vs.* 100%).

^18^F-PSMA-1007 PET/CT demonstrates a superior ability to detect well- or moderately differentiated HCCs compared to ^18^F-FDG and its sensitivity in detecting HCC was independent of tumor differentiation. This provides a new perspective, and these findings may be elucidated by the differing mechanisms of tracer uptake. PSMA is overexpressed in the neovascular endothelial cells of tumors rather than within HCC cells (31). A PSMA-targeting tracer can circumvent the highly heterogeneous avidity exhibited by other tracers targeting the tumor directly. Therefore, the positive incidence of ^18^F-PSMA-1007 PET/CT detection for HCC may not have a direct relationship with the histological grade of HCC cells. Chen et al. has reported that peritumoral/vascular expression of PSMA is greatly associated with grade 3 HCC (5/6, 83.3%) but can also be observed in grade 2 HCC (10/15, 66.7%). This was associated with the clinicopathological characteristics of HCC (36). As such, ^18^F-PSMA-1007 PET/CT can make up for the deficiencies of ^18^F- FDG PET/CT in the detection of low-grade HCC.

The sensitivity of ^18^F-PSMA-1007 PET/CT was not correlated with those clinical and pathological features, such as cirrhosis, AFP levels, tumor number, MVI, or histologic grade.

This result is inconsistent with prior research; Chen et al. reported that HCCs, arising in the setting of cirrhosis (9/10, 90.0%), show a significantly increased peritumoral/vascular PSMA expression compared with non-cirrhotic HCCs (6/12, 50%) (p<0.05) (36). However, it is associated with the size of intrahepatic lesions. ^18^F-PSMA-1007 PET/CT was capable of detecting more small HCC lesions (15 of 24, ≤ 2 cm in diameter) than ^18^F-FDG (6 of 24) in the present cohort, which is consistent with previous studies that consider ^18^F-FDG is not an ideal tracer for small HCCs (37, 38).

The SUV_mean_ of normal liver parencchyma (L) in ^18^F-PSMA-1007 PET/CT was found to be higher than that observed with ^18^F-FDG, indicating notable uptake of ^18^F-PSMA-1007 in normal liver tissue. This observation can be attributed to the distinct biodistribution of ^18^F-PSMA-1007 compared to other radiopharmaceuticals, characterized by its higher lipophilicity. Therefore, ^18^F-PSMA-1007 may be absent in the ureters and bladder due to its predominantly hepatic clearance mechanism (39); this property theoretically renders ^18^F-PSMA-1007 less suitable for HCC imaging compared to other targeting agents. However, in our study, both the SUV_mean_ of intrahepatic lesions (T) and the T/L ratio for ^18^F-PSMA-1007 PET/CT were significantly higher relative to ^18^F-FDG. This is inconsistent with previous similar studies. Gündoğan et al. observed no statistical significance in terms of T/L ratios between ^18^F-FDG and ^68^Ga-PSMA PET/CT (23).

The SUV_mean_ of the abdominal aorta (A) and the right medial gluteal muscle (M) in ^18^F-PSMA-1007 PET/CT were considerably lower than that recorded for ^18^F-FDG across all patients. Additionally, the T/A, and T/M ratios were significantly higher in the context of ^18^F-PSMA-1007 compared to ^18^F-FDG. This finding aligns with the study by Gündoğan et al., which reported a significantly higher T/A and T/M ratios for ^18^F-PSMA-1007 compared to ^18^F-FDG. The results indicated that the uptake level of ^18^F-PSMA-1007 was superior to that of ^18^F-FDG in intrahepatic lesions, while the background uptake of PSMA in blood and muscle were lower. This characteristic might render ^18^F-PSMA-1007 more suitable for HCC imaging than ^18^F-FDG.

Several limitations were noted in the present study. The relatively small sample size in this study limits the reliability of the subgroup analysis results (e.g., based on histologic grade or size grouping), suggesting the need for future multicenter, large-sample studies to further validate these findings. Pathological biopsies were not performed on all lesions, which, whereas not always practical or necessary, may introduce latent bias due to the absence of pathological data in 10 HCC patients diagnosed solely on non-invasive criteria. Additionally, the study cohort consisted of a limited number of patients with suspected HCC who consented to undergo both ^18^F-PSMA-1007 and ^18^F-FDG PET/CT examinations, resulting in an inevitable selection bias.

## Conclusions

Compared to ^18^F-FDG as a PET/CT radiopharmaceutical, ^18^F-PSMA-1007 has tremendous potential value in diagnosing^18^F-FDG PET/CT negative suspected HCC patients. Furthermore, when the uptake of ^18^F-FDG and ^18^F-PSMA-1007 was compared in positive lesions, ^18^F-PSMA-1007 PET/CT exhibited higher values of SUV_max_, T/L, T/A, and T/M. Moreover, as first-line therapy for locally advanced and metastatic HCC consists of a combination of immunotherapy and anti-neoangiogenic treatment (1), PET/CT with PSMA-radioligands may serve as a valuable tool to predict the results of therapy and assess the response to ongoing treatment.

## Data Availability

The original contributions presented in the study are included in the article/supplementary material. Further inquiries can be directed to the corresponding author.
